# Modeling the spatial distribution of Culicoides species (Diptera: Ceratopogonidae) as vectors of animal diseases in Ethiopia

**DOI:** 10.1038/s41598-022-16911-y

**Published:** 2022-07-28

**Authors:** Eyerusalem Fetene, Getachew Teka, Hana Dejene, Deresegn Mandefro, Tsedale Teshome, Dawit Temesgen, Haileleul Negussie, Tesfaye Mulatu, Megarsa Bedasa Jaleta, Samson Leta

**Affiliations:** 1 College of Veterinary Medicine and Agriculture, Addis Ababa University, P. O. Box 34, Bishoftu, Ethiopia; 2 Faculty of Agriculture and Veterinary Science, Ambo University, P.O. Box 19, Ambo, Ethiopia; 3 National Animal Health Diagnostic and Investigation Centre (NAHDIC), P. O. Box 4, Sebeta, Ethiopia

**Keywords:** Entomology, Ecology

## Abstract

*Culicoides* biting midges (Diptera: Ceratopogonidae) are the major vectors of bluetongue, Schmallenberg, and African horse sickness viruses. This study was conducted to survey *Culicoides* species in different parts of Ethiopia and to develop habitat suitability for the major *Culicoides* species in Ethiopia. *Culicoides* traps were set in different parts of the country from December 2018 to April 2021 using UV light Onderstepoort traps and the collected Culicoides were sorted to species level. To develop the species distribution model for the two predominant *Culicoides* species, namely *Culicoides imicola* and *C. kingi*, an ensemble modeling technique was used with the Biomod2 package of R software. KAPPA True skill statistics (TSS) and ROC curve were used to evaluate the accuracy of species distribution models. In the ensemble modeling, models which score TSS values greater than 0.8 were considered. Negative binomialregression models were used to evaluate the relationship between *C. imicola* and *C. kingi* catch and various environmental and climatic factors. During the study period, a total of 9148 *Culicoides* were collected from 66 trapping sites. Of the total 9148, 8576 of them belongs to seven species and the remaining 572 Culicoides were unidentified. The predominant species was *C. imicola* (52.8%), followed by *C. kingi* (23.6%). The abundance of these two species was highly influenced by the agro-ecological zone of the capture sites and the proximity of the capture sites to livestock farms. Climatic variables such as mean annual minimum and maximum temperature and mean annual rainfall were found to influence the catch of *C. imicola* at the different study sites. The ensemble model performed very well for both species with KAPPA (0.9), TSS (0.98), and ROC (0.999) for *C. imicola* and KAPPA (0.889), TSS (0.999), and ROC (0.999) for *C. kingi*. *Culicoides imicola* has a larger suitability range compared to *C. kingi*. The Great Rift Valley in Ethiopia, the southern and eastern parts of the country, and the areas along the Blue Nile and Lake Tana basins in northern Ethiopia were particularly suitable for *C. imicola*. High suitability for *C. kingi* was found in central Ethiopia and the Southern Nations, Nationalities and Peoples Region (SNNPR). The habitat suitability model developed here could help researchers better understand where the above vector-borne diseases are likely to occur and target surveillance to high-risk areas.

## Introduction

African horse sickness (AHS) and bluetongue (BT), as well as Schmallenberg virus (SBV), are among the best-known animal diseases transmitted by adult female Culicoides biting midges^1–3^. AHS is a non-contagious viral disease of equids^1,3^ while BT, and SBV affect ruminants^1,4^. AHS disease is endemic in many parts of Africa, especially in the central and eastern parts of the continent, where it periodically makes short excursions beyond these areas^5,6^. BT has also historically been endemic in many countries located between 40° north and 35° south latitude^7^. However, since 1998, an unprecedented spread of BT has been observed in the Mediterranean basin^8^. SBV is a recent arboviral disease known to cause abortions, stillbirths, and congenital malformations in cattle, sheep, and less commonly, goats^9^. SBV was first detected in Germany in 2011 and since then the disease has spread to almost all European countries^4^. The disease has also been reported outside Europe from Ethiopia^10^.

Ethiopia experiences serious and repeated outbreaks of AHS every year. From 2007 to 2010, about 737 outbreaks were reported in different parts of the country^11^. However, the status of BT and SBV is not well understood, which may be due to misdiagnosis of the diseases with other common ruminant diseases such as foot and mouth disease (FMD), peste des petits ruminants (PPR), lumpy skin disease, and sheep and goat pox, which cause similar clinical symptoms^12^. However, there are some serological reports of BT^13,14^. For SBV, although there is no molecular evidence or virus isolation, a high apparent seroprevalence of 56.6% has been reported^10^.

*Culicoides* biting midges (Diptera: Ceratopogonidae) is a genus of the smallest blood-sucking flies, which measures up to 3 mm in size. The genus has a worldwide distribution, except in Antarctica and New Zealand, and it has more than 1400 known species^15,16^. *Culicoides* are known to transmit a wide range of pathogens. More than 50 arboviruses belonging to the Bunyaviridae, Reoviridae, and Rhabdoviridae families have been isolated from various Culicoides species^17^.

Culicoides breed in a variety of habitats and tend to stay near their hosts, including in and around farms, decaying vegetation, manure, pond edges, and moist soils. Female *Culicoides* often seek blood as a protein source for the development of their eggs. Therefore, they often bite their hosts such as amphibians, birds, and mammals, including humans and domestic animals to feed on their blood^18^.

Several studies have investigated the occurrence and species composition of *Culicoides* species throughout the world^19–23^. Some have predicted the potential current and future geographic distribution of *Culicoides* midges using different climatic and environmental variables^24–29^. To date, several *Culicoides* species have been detected in Ethiopia, including *C. milnei*, *C. zuluensis*, *C. imicola*, *C. neavei*, *C. fulvithorax*, and *C. isioloensis*^30^ and *C. fuscicaudae*^31^.

For effective risk management, it is essential to know the species composition and abundance of Culicoides populations in an area^32^. This study is, therefore, aimed to survey *Culicoides* species in different parts of Ethiopia and to develop habitat suitability for the prevalent species.

## Materials and methods

### Culicoides collection sites

**Figure 1:**
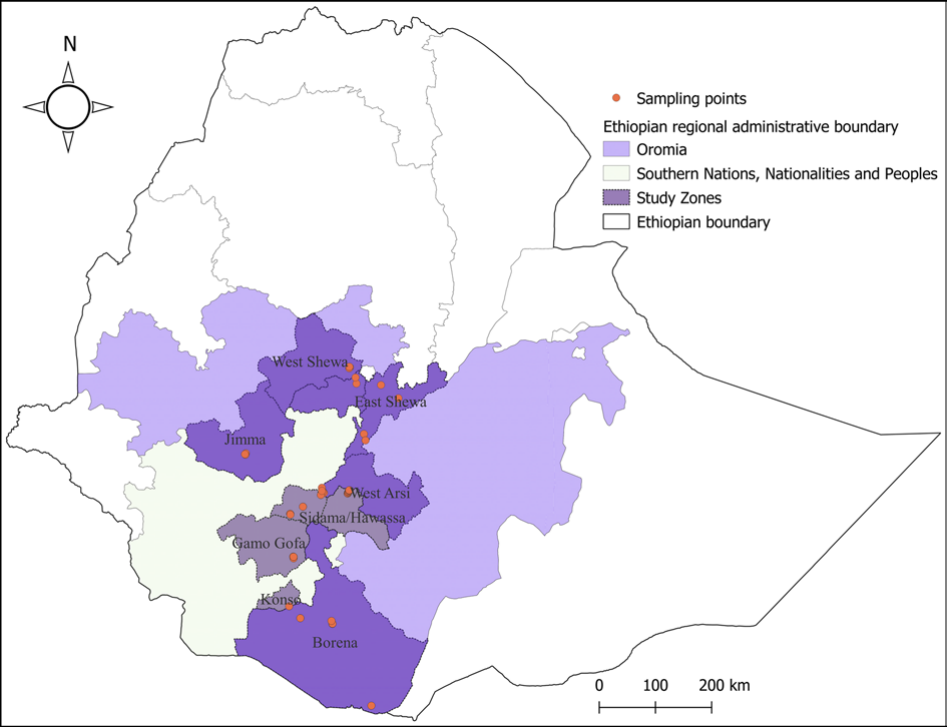
Map of *Culicoides* collection sites. The map was created using QGIS software v 3.22.2 (https://qgis.org/).

The current study was conducted in different parts of Ethiopia belonging to two regions. Hawassa town, Gamo Gofa, Konso and Wolayita in the South Nation Nationalities and People Region (SNNPR) and Jimma, Oromia Special Zone, East Shewa and Borena in the Oromia Region (Fig. 1). The areas were selected based on previous reports of Culicoides-borne diseases, namely AHS, bluetongue and SBV. The study is in accordance with relevant guidelines and regulations.

### Culicoides collection and identification

*Culicoides* trapping was conducted from December 2018 to April 2021, consisting of two rounds. Sampling of the first round was conducted from December 2018 to April 2019 in Hawassa town, Gamo Gofa, Konso, Wolayita, East Shewa and Borena zones, while the second round was conducted from November 2020 to April 2021 in East Shewa, Jimma and Oromia Special zones. *Culicoides* were collected using UV light/suction traps developed by Onderstepoort Veterinary Institute (OVI, South Africa) and powered by a 12 V car battery. Trap locations were selected to ensure that they were locations conducive to the reproduction of *Culicoides* species. These include areas near water bodies, wetlands, livestock farms, and/or equine stables (Table 1). The location of each trap site was obtained with Global Positioning System (GPS). Traps were set from dusk (6:00 p.m.) to dawn (6:00 a.m.) and were placed both outdoors and indoors. Traps were hung from tree branches or building eaves at a height of 1.5–2 m above the ground. Insects were collected overnight in the cups of the traps and retrieved the next morning. All catches were transported to a local laboratory and placed in a − 4 °C refrigerator for 15 min to tranquillize insects. *Culicoides* species were first separated from other insects under a stereomicroscope, then transferred to an insect collection tube containing 70% ethanol, and finally transported to the entomology laboratory at the National Animal Health Diagnostic and Investigation Centre (NAHDIC) for species identification.

**Table 1:** Total number of traps in each zone.

Zones	Total number of traps	Total number of traps near animal farm		Total number of traps near water bodies
		Indoor (inside farms)	Outdoor (outside farms)	
**First round sampling**				
East Shewa	13	–	6	7
Hawassa town	10	–	10	–
Gamo Gofa	8	1	2	5
Wolayita	8	6	2	–
Borena	6	–	4	2
Konso	1	–	–	1
**Second round sampling**				
East Shewa	4	–	4	–
Oromia Special Zone	10	–	10	–
Jimma	6	–	6	–
Total	66	7	44	15

Species identification and enumeration were performed by observation of morphological features of *Culicoides* under a stereomicroscope. Identification was made according to previously published identification keys^31,33,34^. Most Culicoides midges have a wing pigmentation pattern and a distribution of wing macrotrichia consisting of grey and white spots; these patterns are unique to each species and can be easily observed under a dissecting microscope. The specimens were mounted on a slide and under the light microscope, morphological features such as shape, size, the number of female spermathecae, and the distance between eyes were observed^33^. Then we observed the ratio of antennae XI/X (length of segment XI divided by the length of segment X) and the shape and size of the 3rd palpal segment. Finally, we compared all the observed features with the images in the IIKC database (Interactive Identification Key for *Culicoides*)^33^.

### Spatial distribution modeling (SDM)

The geographic distribution of the two most common *Culicoides* spp. was predicted using an ensemble modeling technique^35^. Species distribution models consists of three main aspects: species occurrence data (dependent variable), layers of environmental variables (independent variables), and a modeling algorithm.

Climate and environmental variables that characterize favorable habitats for *Culicoides* were selected based on a literature review of presence and abundance models^25,36–38^. Minimum, mean, and maximum temperature, precipitation, solar radiation (kJ m^−2^ day^−1^), wind speed (ms^−1^), water vapor pressure (kPa) and altitude were downloaded from the WorldClim database version 2 (http://worldclim.org/).

Land cover data was downloaded from the European Space Agency’s GlobCover Portal (http://due.esrin.esa.int/page_globcover.php). Livestock distribution data was downloaded from the website of FAO livestock systems (http://www.fao.org/livestock-systems/). Soil type was downloaded from (https://www.iiasa.ac.at/web/home/research/researchprograms/water/HWSD.html). All data layers were projected in the same projection system with a spatial resolution of 2.5 arc minutes using QGIS 3.4.1.

### Data analysis

Ensemble modeling technique using biomod2^35^ package of R software (http://cran.r-project.org/web/packages/biomod2/index.html) was used to develop the SDM. The package uses ten different methods: general linear models (GLM), general boosted models (GBM, also called boosted regression trees), general additive models (GAM), classification tree analysis (CTA), artificial neural networks (ANN), surface range envelope (SRE), flexible discriminant analysis (FDA), multiple adaptive regression splines (MARS), random forests (RF), and maximum entropy (MAXENT)^35^. Because the above ten modeling techniques require both absence and presence data to determine the suitability range of species, pseudo-absence data were generated using Surface Range Envelope (SRE). The data was split into two parts, 80% was used to train the model and 20% was used to test model performance. Models were evaluated using the results of threefold cross-validation in 30 models (10 techniques × 3 replicates).

The performance of models were evaluated using the true-skill statistic (TSS), the area under the receiver-operating characteristic curve (ROC) curve, and the Cohen's kappa statistic (Kappa). TSS is a threshold-dependent evaluation (sensitivity + 1, specificity − 1), with values closer to one indicating model accuracy^39^. For ensemble modeling, only models which score TSS values greater than 0.8 were considered. AUC scores range from 0 to 1, with models with scores above 0.5 providing better predictions than random draws^40^.

Since there is overdispersion in our multivariable Poisson model, a negative binomial regression models were used to assess the relationship between *C. imicola* and *C. kingi* catch with various environmental and climatic factors including agro-ecological zonation, habitat, livestock density, soil type, mean annual minimum and maximum temperatures, annual precipitation, solar radiation, wind speed, land cover and water vapour pressure. Univariable negative binomial regression models were first developed and only variables which are significant on univariable analysis were used to develop the multivariable negative binomial regression models. Multicollinearity among explanatory variables was checked using variance inflation factor (VIF) analysis, with the “*vifstep”* command in the “*usdm”* package of R. Variables which have VIF values less than or equal to 10 were considered in the analysis^41^. Mean annual maximum temperature was removed from *C. imicola* model due to collinearity and mean annual minimum temperature, altitude and solar radiation were removed from *C. kingi* model due to multicollinearity.

### Ethics approval and consent to participate

Ethical approval was obtained from ethics committee of the College of Veterinary Medicine and Agriculture of Addis Ababa University. Written informed consents were obtained from all households who participated in the study.

## Result

### Entomological survey

During the study period, a total of 9148 *Culicoides* were collected from 66 trapping sites. Of the total 9148, 8576 of them belongs to seven species and the remaining 572 *Culicoides* were unidentified. Of the seven species identified, *C. imicola* was the most abundant species with 4830 (52.8%), followed by *C. kingi* with 2160 (23.6%), *C. milnei*, *C. schultezi* and *C. Zuluensis*, which accounted for 9%, 6.8%, and 1.4% of the catch, respectively (Table 2). Most *Culicoides* were caught in Jimma zone (28.5%), followed by Hawassa town (19.4%) and Oromia special zone (17.4%).

**Table 2:** *Culicoides* species collected across the study sites.

Culicoides species	Collection sites								Number and %
	Hawassa town	East Shewa	Gamo Gofa	Wolayita	Borena	Segen Valley	Oromia Special Zone	Jimma	
*C. imicola*	1512	775	228	113	133	196	696	1177	4830 (52.8%)
*C. kingi*	142	4	23	2	419	316	328	926	2160 (23.6%)
*C. milnei*	–	–	–	–	–	–	289	533	822 (9%)
*C. schultzei*	28	20	–	–	80	488	–	–	616 (6.8%)
*C. zuluensis*	89	41	–	–	–	–	–	–	130 (1.4%)
*C. pycnostictus*	2				16				18 (0.2%)
Others	5	16	98	17	161	–	275	–	572 (6.2%)
Total (%)	1778 (19.4%)	856 (9.3%)	349 (4%)	132 (1.4%)	809 (9%)	1000 (11%)	1588 (17.4%)	2636 (28.5%)	9148 (100%)

Most Culicoides were collected in the vicinity of the animal pen 6926 (75.7%), 1000 Culicoides were collected near rivers, which constitute 11% of the catch (Table 3).

**Table 3:** Culicoides species collected from different habitats.

Culicoides species	Indoor	Lake shore	Animal pen	Near pond	Outdoor on field	River	Total count
*C. imicola*	59	203	4251	49	72	196	4830
*C. kingi*	1	64	1409	171	199	316	2160
*C. milnei*	–	–	822	–	–	–	822
*C. schultzei*	–	7	121	–	–	488	616
*C. zuluensis*	–	20	110	–	–	–	130
*C. pycnostictus*	–	–	18	–	–	–	18
Unidentified	8	100	195	–	143	–	572
Total (%)	68 (0.74%)	394 (4.3%)	6926 (75.7%)	220 (2.4%)	414 (4.5%)	1000 (11%)	9148 (100%)

### Factors associated with *Culicoides imicola* and *Culicoides kingi* occurrence

The impact of different environmental and climatic variables on the abundance of *C. imicola* and *C. kingi* was evaluated using multivariable negative binomial regression (Tables 4, 5). Agro-ecological zonation, habitat, soil type, mean annual minimum temperature, altitude, and water vapour pressure were found to have a significant effect on the number of *C. imicola* catches (Table 4). *Culicoides imicola* catches were higher near animal pen and in nitisols and vertisols soil types. When mean annual minimum temperature and altitude increases, *C. imicola* catches decrease (Table 4).

**Table 4:** Factors associated with *C. imicola* occurrence using multivariate negative binomial regression analysis.

Factors	Number of traps	Total number of *C. imicola* collected	Mean number of *C. imicola* collected (mean ± SD)	Negative binomial regression coefficient	p-value
**Agro-ecological zonation**					
Arid	22	2051	93.2 ± 98.3	Ref	
Semi-Arid	16	523	32.7 ± 104.1	− 1.048	0.0103
Sub-humid	12	2256	188.0 ± 190.6	0.701	0.114
**Habitat**					
Animal pen	28	4205	150.2 ± 156.8	Ref	
Indoor	6	59	9.8 ± 12.5	NA	NA
Outdoor	2	72	36.0 ± 3.0	− 45.19	0.000
Water shore^a^	14	494	35.3 ± 47.2	− 0.80795	0.091
**Soil type**					
Andosols	4	183	45.8 ± 23.4	Ref	
Fluvisols	18	1661	92.3 ± 48.8	1.652	0.001
Leptosols	10	461	46.1 ± 13.2	1.021	0.177
Luvisols	5	212	42.4 ± 146.8	NA	NA
Nitisols	2	548	274 ± 52.3	26.857	0.000
Vertisols	11	1765	160.5 ± 113.4	0.828	0.561

	Mean ± SD	Minimum	Maximum		
Mean annual minimum temperature (°C)	13.72 ± 2.37	9.1	18.3	− 1.613	0.000
Altitude (masl)	1572 ± 311.4	843	2390	− 0.051	0.001
Water vapour pressure	1.58 ± 0.17	1.12	1.87	125.453	0.000

^a^Water shore includes river side, near pond, and lake shore.

**Table 5:** Factors associated with *C. kingi* occurrence using multivariate negative binomial regression analysis.

Factors	Number of traps	Total number of *C. kingi* collected	Mean number of *C. kingi* collected (mean ± SD)	Negative binomial regression coefficient	p-value
**Agro-ecological zonation**					
Arid	16	721	45.1 ± 77.9	Ref	
Semi-Arid	4	184	46.0 ± 72.2	4.885	0.000
Sub-humid	5	1255	251.0 ± 300.1	10.980	0.000
**Habitat**					
Animal pen	14	1409	100.6 ± 212.4	Ref	
Indoor	1	1	1.0 ± 0	− 6.987	0.000
Outdoor	2	199	99.5 ± 9.5	9.950	0.000
Water shore^a^	8	551	68.9 ± 107.8	3.754	0.000
**Soil type**					
Andosols	1	4	4 ± 0	Ref	
Fluvisols	13	401	30.8 ± 8.6	3.182	0.000
Leptosols	1	320	320 ± 0	7.652	0.000
Luvisols	2	179	89.5 ± 43.2	2.735	0.000
Nitisols	2	328	164 ± 34	− 1.033	0.541
Vertisols	4	928	232 ± 59.8	0.691	0.589

	Mean ± SD	Minimum	Maximum		
Mean annual maximum temperature (°C)	27.6 ± 1.98	23.05	30.98	− 1.767	0.000

^a^Water shore includes river side, near pond, and lake shore.

Agro-ecological zonation of trapping sites, habitat, soil type and mean annual maximum temperature are significantly related to the occurrence of *C. kingi*. The catches of *C. kingi* were higher in humid areas, and in leptosols, nitisols and vertisols soil types. Mean annual maximum temperature is found to have a antagonistic effect with the *C. kingi* catches, as mean annual maximum temperature increases *C. kingi* catches decreases (Table 5).

### Species distribution modeling

We chose to model the distribution of the two most common *Culicoides* species, *C. imicola* and *C. kingi*. The ensemble model performed very well for both species with (KAPPA (0.9), TSS (0.98), and ROC (0.999) for *C. imicola* and (KAPPA (0.889), TSS (0.999), and ROC (0.999) for *C. kingi*.

**Figure 2:**
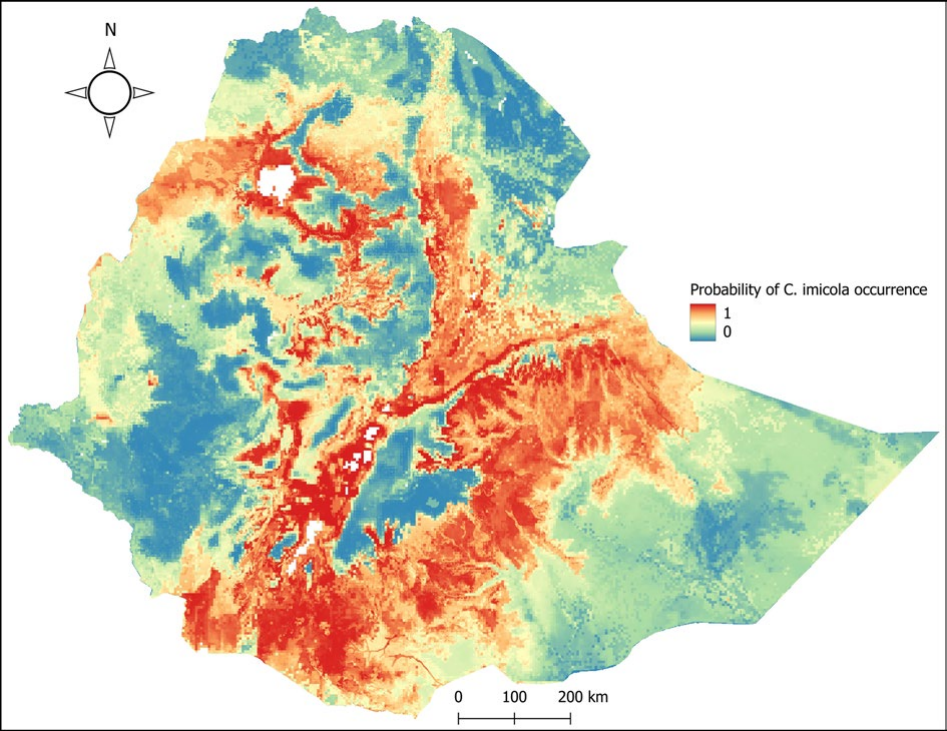
Probability of *C. imicola* occurrence in Ethiopia. Highly suitable areas are represented with red color while unsuitable areas are represented using blue colour. The map was created using QGIS software v 3.22.2 (https://qgis.org/).

*Culicoides imicola* has a wider suitability range compared to *C. kingi*. The Great Rift Valley in Ethiopia and southern and eastern Ethiopia have a high suitability range for *C. imicola*. Suitability of *C. imicola* is also high in northern Ethiopia, particularly along the Blue Nile and Lake Tana catchments. Central Ethiopia has a patchy suitability range. The model predicts Gambela, Benshangul Gumuz, western parts of Oromia, Tigray, Afar, and Somali region as moderately suitable (Fig. 2).

**Figure 3:**
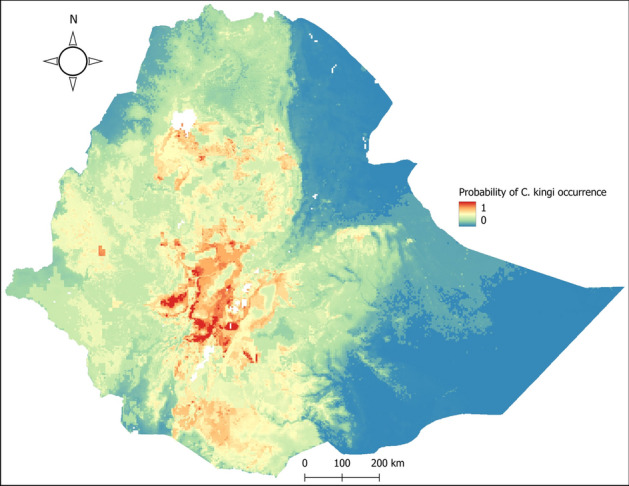
Probability of *C. kingi* occurrence in Ethiopia. Highly suitable areas are represented with red color while unsuitable areas are represented using blue color. The map was created using QGIS software v 3.22.2 (https://qgis.org/).

A high suitability range for *C. kingi* was observed in central Ethiopia and in SNNPR. The ensemble model predicted the other parts of the country as moderately suitable, with the exception of the Afar and Somali regions, for which the model predicted lower suitability (Fig. 3).

According to the results of this ensemble modeling, the distribution of *C. imicola* depends mainly on soil type (15.7%), altitude (12.5%), livestock distribution (12.5%), solar radiation (12.1%), and mean annual minimum temperature (11.8%). For *C. kingi*, they are wind speed (43.4%), soil type (10.8%), altitude/elevation (10.6%), and vapor pressure (8.8%) (Table 6).

**Table 6:** Variables contribution in the predicted distribution of *C. imicola* and *C. kingi.*

Variables	Contribution (%)	
	*C. imicola*	*C. kingi*
Soil type	15.8	10.8
Altitude	12.5	10.6
Livestock distribution	12.5	7.4
Solar radiation	12.1	6.4
Mean annual minimum temperature (°C)	11.8	1.3
Annual precipitation (mm)	9.7	3.3
Land cover	8.9	3.3
Water vapor pressure	7.0	8.8
Wind speed	5.0	43.4
Mean annual maximum temperature (°C)	4.8	4.7

## Discussion

AHS, BT bluetongue and SBV are economically important Culicoides-borne viral diseases affecting equids and ruminants. The importance of these arboviral diseases derives from their very wide geographic distribution, potential for rapid spread, and large economic impact^1,2^. A considerable number of studies reported widespread occurrence of these diseases in Ethiopia. Demissie^42^ reported AHS from Gamo Gofa, Wolaita and Hadiya zones of SNNPR. Zeleke et al.^43^ reported the occurrence of AHS in southern (Awassa, Hossana, Wondogenet, and Hagereselam), western (Jimma, Bedelle, Nekemte, Horroguduru, and Chaliya), and central (Bishoftu, Meki, Zeway, Filtimo, and Bekejo) Ethiopia. Ayelet et al.^44^ reported AHS from Ada’a, Bahir Dar, Mecha, Dangla, Jimma and Sodo and Aklilu et al.^11^ reported AHS from central Ethiopia. Gizaw et al.^13^ reported the presence of group-specific antibodies against bluetongue virus from Adami Tulu, Amibara, Areka, Arsi Negelle, Bene Tsemay, Doyo Gena, G/Mekeda, Fafan, and Jinka. Abera et al.^14^ reported the presence of bluetongue antibodies from Jimma, Bonga and Bedelle and Gulima^45^ reported the presence of bluetongue antibodies from Amhara regional state in northern Ethiopia. There is only one study on the sero-prevalence of SBV in Ethiopia. The author reported a very high apparent seroprevalence of 56.6%^10^.

These diseases are transmitted by females of several species of midges belonging to the large genus Culicoides (Diptera: Ceratopogonidae) (which includes more than 1300 described species worldwide^46,47^. In this study, morphological identification confirmed the presence of seven species in Ethiopia. In the current study, various Culicoides species were collected, of which *C. imicola* was the largest number 4830 (52.8%). Similar to these results, entomological surveys carried out in many sub-Saharan African countries show that *C. imicola* is the dominant species^22,23,48–50^. A previous study by Mulatu and Hailu^30^, reported the presence of *C. imicola, C. milnei*, *C. neavei*, *C. zuluensis*, *C. fulvithorax* and *C. isioloensis* in western parts of Ethiopia and Khamala and Kettle^31^ reported the presence of *C. fuscicaudae*. This study identified three species of *Culicoides* that had not been previously described in Ethiopia, including *C. kingi, C. schultzei* and *C. pycnostictus.*

Multivariable negative binomial regression models were used to model the impact of various environmental and climatic factors on *C. imicola* and *C. kingi* catch. For both species, significantly higher catch was obtained in the subhumid agro-ecological zone. This finding suggests that Culicoides species require breeding habitat with high relative humidity^51^. In the current study, higher numbers of Culicoides were caught in traps placed near animal pens. This result is consistent with Riddin et al.^52^ that reported high Culicoides catches near horse barns. The abundance of Culicoides near animal pens is mainly due to the presence of suitable breeding sites represented by moist soil sites, leaking animal watering troughs, and pond edges contaminated with feces^53^.

Soil type appears to be very important in determining the distribution and abundance of *Culicoides*^23,54,55^. According to the studies, the largest numbers of *Culicoides* were found in areas with a high, moisture-retentive clay soil, whilst the lowest numbers were encountered in rapidly draining sandy soils. In this study, *C. imicola* is mainly collected from nitisols and vertisols soil type, these soils types are clay-rich and characterized by their high moisture retention capacity^56,57^. *C. kingi* was mainly collected from diverse soil types including leptosols, nitisols and vertisols soil types. Leptosols are mountain soils known by their high waterlogging capacity^58^. These soil types are believe to create suitable breeding sites for different Culicoides species.

The present study shows that climatic variables to be an important determinants of *Culicoides* catches, temperature being the most important determinant for both species. Studies demonstrated that, *Culicoides* activity generally declines or even ceases at low temperatures, and high temperatures. Temperature ≥ 40 °C could be lethal^38^. Foxi et al.^53^ also reported relatively poor tolerance of *Culicoides* to lower temperatures. Eventhough it is not evident in our multivariable negative binomial regression models, previous study by Gusmão et al.^21^ suggests that persistent or heavy rain can create conditions for biting midges to proliferate but it can also be a barrier to the activity of adult (winged) biting midges and prevent them from flying. Rain can prevent adults from leaving their shelters and could affect catch.

Various climatic and environmental factors were used to model the species distribution of *C. imicola* and *C. kingi*. Soil type, altitude/elevation, livestock distribution, solar radiation, and mean minimum annual temperature were the most important variables for the *C. imicola* model. Wind speed, soil type, altitude/elevation, and vapor pressure were the variables that contributed most to the model for *C. kingi*. Although there is no previous information on the climatic requirements of *C. kingi*, numerous studies have examined the role of various climatic and environmental factors on the distribution and abundance of *C. imicola*. Global ensemble modeling of *C. imicola* by Leta et al.^29^ reported temperature covariates contributing 64% to their model. This is supported by Veronesi et al.^59^ which showed that temperature can affect fecundity, hatching, and survival of *C. imicola*. When reared at a higher temperature (28 °C), *C. imicola* exhibited higher variability in fecundity and lower hatch rates, and the mean emergence rate from pupae was highest at 20 °C. The distribution of *C. imicola* is probably directly limited by its relatively low tolerance to lower temperatures^60^. As temperatures rise, adults hatch and populations gradually increase to reach a peak in abundance in spring or summer, depending on the site, which is a function of spring temperatures and summer drought. Because temperature shortens larval development time and the time between two blood meals, thus increasing laying frequency, which has a positive effect on population dynamics (and their growth), we expected that temperature would have a significant effect on abundance^24^.

Our study also showed that solar radiation and livestock distribution are influential variables in the spread of *C. imicola.* According to Conte et al.^61^, intense solar radiation on *C. imicola* larval habitat combined with high nighttime temperatures accelerates larval development, resulting in multiple generations/season. The importance of livestock as a source of blood meals for *C. imicola* is well established^62^. *Culicoides imicola* is a bloodsucking insect that tends to feed on blood and breed near livestock and humans. The frequency of contact between *Culicoides* and vertebrate hosts is closely related to the multiplication of the pathogen and the risk of transmission^62,63^.

In this study, *C. imicola* was found to have a larger suitable range compared to *C. kingi*. Globally, *C. imicola* is widespread in tropical and subtropical regions of Africa, the southern part of Europe, and some parts of Asia^64^. The model describes that all regions have a small to large range of suitable areas, with Oromia and SNNPR regions having a larger range of suitable areas. High suitability for *C. imicola* was also demonstrated in the Amhara region, particularly adjacent to the Blue Nile basin and Lake Tana, in southern Afar, and in areas of the Somali region adjacent to Oromia. The results of the current model overlap in many ways with the previously published model of Leta et al.^29^. The current model emphasizes at national level by elaborating the distribution of *C. imicola* and *C. kingi* suitability in different regions of the country.

In conclusion, the entomological study shows the occurrence of *C. imicola* and *C. kingi* in different parts of Ethiopia, with C. imicola predominating. The widespread occurrence of these species indicates a higher risk of SBV, BT and AHS in different parts of Ethiopia. The models could help to understand the risk of introduction and spread of SBV, BT, and AHS.

## Data Availability

The datasets generated during and/or analyzed during the current study are available from the corresponding author up on request.
