# Author Correction: Modeling the spatial distribution of Culicoides species (Diptera: Ceratopogonidae) as vectors of animal diseases in Ethiopia

**DOI:** 10.1038/s41598-022-18764-x

**Published:** 2022-09-02

**Authors:** Eyerusalem Fetene, Getachew Teka, Hana Dejene, Deresegn Mandefro, Tsedale Teshome, Dawit Temesgen, Haileleul Negussie, Tesfaye Mulatu, Megarsa Bedasa Jaleta, Samson Leta

**Affiliations:** 1 College of Veterinary Medicine and Agriculture, Addis Ababa University, P. O. Box 34, Bishoftu, Ethiopia; 2 Faculty of Agriculture and Veterinary Science, Ambo University, P.O. Box 19, Ambo, Ethiopia; 3 National Animal Health Diagnostic and Investigation Centre (NAHDIC), P. O. Box 4, Sebeta, Ethiopia

Correction to: *Scientific Reports* 10.1038/s41598-022-16911-y, published online 28 July 2022

The original version of this Article contained errors, where it was unclear that three identification keys were used for the identification of the species.  

To correct this, in the Materials and methods section, under the subheading ‘Culicoides collection and identification’,

“Species identification and enumeration were performed by observation of morphological features of Culicoides under a stereomicroscope. Most Culicoides midges have a wing pigmentation pattern and a distribution of wing macrotrichia consisting of grey and white spots; these patterns are unique to each species and can be easily observed under a dissecting microscope.”

now reads:

“Species identification and enumeration were performed by observation of morphological features of Culicoides under a stereomicroscope. Identification was made according to previously published identification keys [31, 33, 34]. Most Culicoides midges have a wing pigmentation pattern and a distribution of wing macrotrichia consisting of grey and white spots; these patterns are unique to each species and can be easily observed under a dissecting microscope.”

Reference 34, listed below, was not included in the original version of the Article:

34. Glick, J. I. Culicoides biting midges (Diptera: Ceratopogonidae) of Kenya. *J. Med. Entomol.* **27**, 85–195 (1990).

As a result of this addition, the subsequent References have been renumbered.

The Article has been corrected.

